# A flow cytometric approach to study the mechanism of gene delivery to cells by gemini-lipid nanoparticles: an implication for cell membrane nanoporation

**DOI:** 10.1186/s12951-015-0125-1

**Published:** 2015-09-29

**Authors:** Marjan Gharagozloo, Amirreza Rafiee, Ding Wen Chen, Marianna Foldvari

**Affiliations:** School of Pharmacy, University of Waterloo, 200 University Avenue West, Waterloo, ON N2L 3G1 Canada

**Keywords:** Non-viral vectors, Nanoparticles, Gemini surfactants, Plasmid DNA, Gene therapy, Flow cytometry, Confocal microscopy, Lipofectamine Plus

## Abstract

**Background:**

Gemini-lipid nanoparticles have been received major attention recently as non-viral delivery systems due to their successful non-invasive gene delivery through tough barriers such as eye and skin. The aim of this study was to evaluate non-viral gene delivery by a series of dicationic gemini surfactant-phospholipid nanoparticles (GL-NPs) and to explore their mechanism of interaction with cellular membranes of murine PAM212 epidermal keratinocytes.

**Methods:**

NPs containing pCMV-tdTomato plasmid encoding red fluorescent protein (RFP) were prepared using 12 different gemini surfactants (m-s-m, with m = 12, 16 and 18C alkyl tail and s = 3 and 7C polymethylene spacer group and 7C substituted spacers with 7NH and 7NCH3) and dioleoylphosphatidylethanolamine helper lipid. RFP gene expression and cell viability status were evaluated using flow cytometry. MitoTracker Deep Red mitochondrial stain and the cell impermeable Sytox red nuclear stain were used as indicators of cell viability and cell membrane integrity, respectively.

**Results:**

No significant viability loss was detected in cells transfected with 18-3-18, 18-7-18, 18-7NH-18, and 18-7NCH3-18 NPs, whereas a significant reduction of viability was detected in cells treated with 12-3-12, 12-7-12, 12-7NH-12, 16-7NH-16, or 16-7NCH3-16 GL-NPs. Compared to Lipofectamine Plus, 18-3-18 GL-NPs showed higher transfection efficiency and comparable viability profile by evaluation using MitoTracker Deep Red in PAM212 cells. Flow cytometric analysis of PAM212 cells stained with Sytox red revealed two cell populations with low and high fluorescent intensity, representing cells with partially-porated and highly-porated membranes, respectively. Additional combined staining with MitoTracker and ethidium homodimer showed that that 18-3-18 GL-NPs disturbed cell membrane integrity, while cells were still alive and had mitochondrial activity.

**Conclusion:**

Taken together, this study demonstrated that 18-3-18 GL-NPs have higher transfection efficiency and comparable viability profile to the commercial Lipofectamine Plus, and the interaction of 18-3-18 GL-NPs with PAM212 cell membranes involves a permeability increase, possibly through the formation of nanoscale pores, which could explain efficient gene delivery. This novel nanoconstruct appears to be a promising delivery system for further skin gene therapy studies in vivo.

## Background

Gene therapy relies on the delivery of therapeutic genes or manipulation of genetic material in cells and tissues for clinical applications. Gene therapy offers a potentially effective solution for disease prevention or treatment, where current therapeutic approaches have failed or demonstrated limited success. Despite the promising success of gene therapy in the treatment of human diseases [1, 2], many challenges still remain to allow gene therapy to be considered as a standard treatment. Either safety or low efficacy of gene transfer is the main difficulty in current gene therapy approaches.

Viruses are natural vehicles to transfer their genes efficiently to host cells, thereby making them a potent tool for gene delivery in therapeutic applications. However, fundamental concerns such as severe immunization, inflammation, and toxicity (oncogenicity) related to random integration of transferred genetic material into host genome reduce their medical application [3]. In recent years, many studies have been devoted to test and develop various biocompatible nanoparticles as a safe alternative to viral delivery systems. Cationic lipid-nucleic acid complexes including lipids or gemini amphiphiles are of particular interest due to their ability to self-assemble into micelles, liposomes or other polymorphic particles.

Recently, *N*,*N*-bis(dimethylalkyl)-α,ω-alkanediammonium surfactants, also called gemini surfactants, having two cationic head groups and two alkyl tails with the general structure [C_m_H_2m+1_(CH_3_)_2_N^+^(CH_2_)_s_N^+^(CH_3_)_2_ C_m_H_2m+1_.2X^−^] (abbreviated as the m-s-m series, where m and s refer to the number of carbon atoms in the alkyl tails and in the polymethylene spacer group, respectively; and X is the counter ion), received major attention as non-viral delivery systems. Most gemini surfactants with diverse chemical structures demonstrate high gene delivery capacity due to the ability to condense plasmid DNA and self-assemble into small particles with a net positive charge, resulting in electrostatic attraction of gemini nanoparticles (NPs) to the anionic cell surface. Structure–activity studies have been carried out to understand the molecular requirements for NPs formation and cellular transfection [4–6]. The interaction with cell membranes facilitates the internalization of gemini NPs into cells, followed by endosomal escape and localization into the nucleus [7–9].

Recent studies in our group have demonstrated successful gene delivery using gemini-lipid nanoparticles (GL-NPs) through tough barriers such as eye and skin [6, 10]. We have previously shown that the 12-s-12 first generation (unsubstituted) gemini surfactant-dioleoylphosphatidylethanolamine (DOPE)-based NPs are effective gene delivery vectors but with efficiency still lower than commercial transfection reagents in keratinocytes [6, 11, 12]. However, their toxicity and transfection efficiency, both in vitro and in vivo, depend on various parameters such as chemical structure of the gemini surfactant, plasmid/gemini charge ratio and the presence of helper lipids. In order to find the most efficient GL-NPs candidates for topical in vivo gene delivery, this study was performed to investigate the transfection efficiency and biocompatibility of 12 different GL-NPs in an epidermal PAM212 keratinocyte cell line. Another aim of this study was also to investigate whether the interaction of GL-NPs with the cells induces cell membrane damage or affects metabolic activity of the cells using flow cytometry. In contrast to traditional colorimetric- or fluorescent-based assays, flow cytometry offers the advantages of detecting several biological parameters of the cells at the same time. It has been shown that cationic nanoparticles, including cationic gold nanoparticles (AuNPs) [13], quantum dots [14], or dendrimers [15, 16] can adhere strongly to the cells and form pores on cell membranes, leading to the exchange of ions and small molecules through the membrane and potentially cause cytotoxicity. In this current report, we observed for the first time that cationic 18-3-18 GL-NPs disrupt the integrity of the cell membranes without interfering with the metabolic activity of the cells. These results have implication for our understanding of the efficiency of the GL-NPs as non-viral drug/gene delivery agents.

## Results

### Particle size of GL-NPs

The size and PDI of the GL-NPs used for the delivery of tdTomato pDNA to PAM212 cells are presented in Table 1. Most GL-NPs made with first generation (unsubstituted) gemini surfactants were in the size range of 100–170 nm, whereas second generation gemini surfactants (substituted in the spacer with imino or aza groups) formed larger particles in the 12 and 18-series. Some exceptions were also noted, e.g. 18-3-18 generated a relatively more heterogeneous population of large particles over 1 µm. The 16-series NPs were small (about 130 nm) regardless of the type of spacer substitution or alkyl tail length. It has been previously shown that the chemical structure of the gemini surfactants has an effect on the size of GL-NPs [11]. In the 12-series gemini surfactants, results showed that changes to the spacer chemical structure significantly affected the particle size, where adding aza (NCH3) group to the spacer significantly increased particle size (12-7NCH3-12 > 12-7NH-12 > 12-7-12). However, such effect was not observed in the 16 and 18 series, where adding imino or aza group to the spacer showed no significant effect on particle size. Increasing the tail length was shown to have a noticeable effect on the particle size of GL-NPs made by m-7-m gemini (18-7-18 > 16-7-16 > 12-7-12). However, this trend was not observed in GL-NPs prepared by m-3-m gemini (18-3-18 > 16-3-16 ≈ 12-3-12), because 18-3-18 gemini produced large particles (1276.97 ± 291.28 nm) compared to the size range of other GL-NPs (104.13 ± 0.68 to 351.20 ± 14.72 nm). The relatively large particle size obtained with 18-3-18 GL-NPs were probably the result of the very low critical micelle concentration (CMC; 13 µM) of 18-3-18 compared to other gemini surfactants [11].

**Table 1:** Hydrodynamic diameter and PDI of GL-NPs used for the delivery of pDNA to PAM212 cells, presented as mean ± standard deviation

Gemini nanoparticle	Average size (nm)	PDI
12-3-12	155.000 ± 2.364	0.393 ± 0.007
12-7-12	104.133 ± 0.680	0.222± 0.003
12-7NH-12	168.333 ± 2.324	0.298 ± 0.005
12-7NCH3-12	293.983 ± 10.372	0.461 ± 0.051
16-3-16	134.133 ± 1.201	0.219± 0.009
16-7-16	126.600 ± 2.291	0.289 ± 0.011
16-7NH-16	126.266 ± 0.493	0.198 ± 0.009
16-7NCH3-16	118.533 ± 1.078	0.277 ± 0.004
18-3-18	1045.267 ± 157.057	0.491 ± 0.159
18-7-18	170.033 ± 4.158	0.158 ± 0.125
18-7NH-18	351.200 ± 14.729	0.345 ± 0.033
18-7NCH3-18	160.266 ± 1.795	0.397 ± 0.010

### GL-NPs treatment and the viability of PAM212 cells

**Fig. 1:**
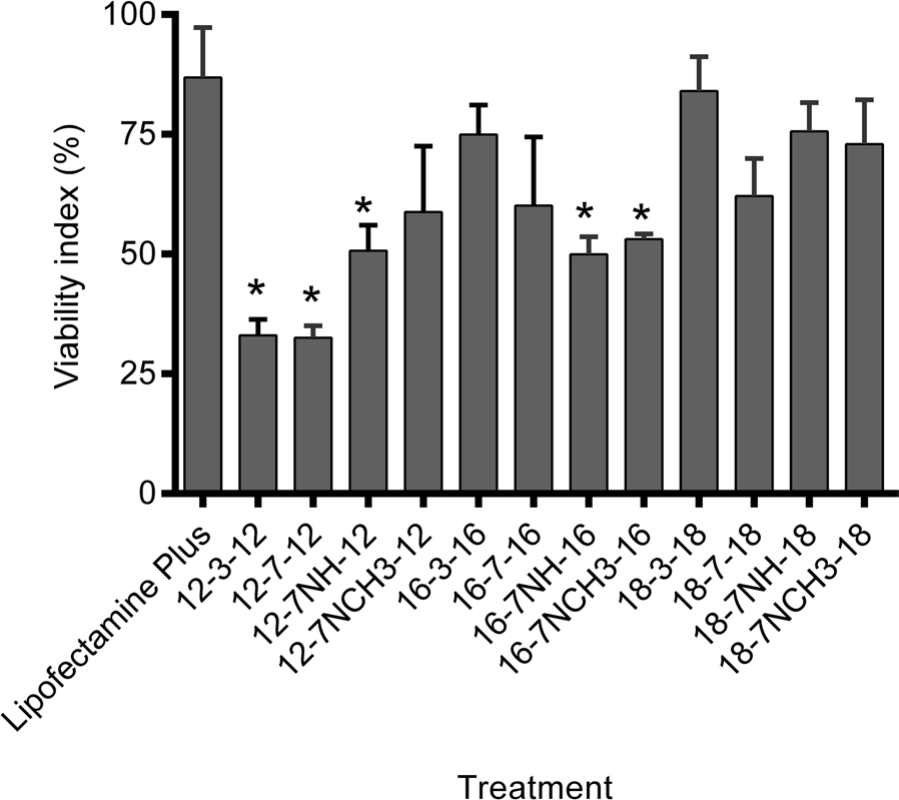
Effect of GL-NPs on the viability of PAM212 cells measured by MitoTracker staining and flow cytometry. Results are expressed as the mean of cell viability index ± standard deviation compared to the untreated control (as 100 %). *Asterisks* represent LSD post hoc statistical significance compared to Lipofectamine Plus (P < 0.05). *Bar charts* represent mean ± SD, n = 4

In the first flow cytometry study, MitoTracker Deep Red (a cell permeant far-red fluorescent dye), that stains live cells with intact [17] mitochondrial trans-membrane potential, was used to assess the viability of PAM212 cells transfected with GL-NPs including the 12, 16 and 18 series and Lipofectamine Plus as a reference transfection agent. In this assay all cells stained positive for MitoTracker were counted as ‘viable’. Results revealed some degree of viability loss in all NPs-treated cells. An insignificant effect on viability was seen with 18-3-18, 18-7-18, 18-7NH-18, and 18-7NCH3-18 NPs and Lipofectamine Plus (Fig. 1), whereas relatively high toxicity (30–50 % viability) was detected in cells treated with 12-3-12, 12-7-12, 12-7NH-12, 16-7NH-16, and 16-7NCH3-16.

### Transfection efficiency of GL-NPs in PAM 212 cells

**Fig. 2:**
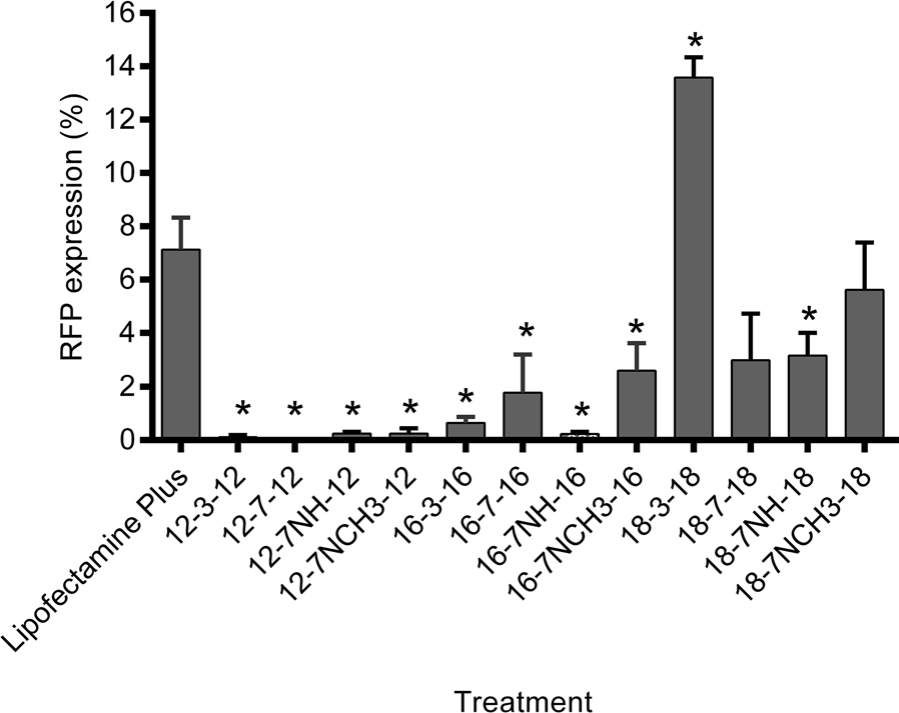
RFP expression in PAM212 cells, transfected GL-NPs and Lipofectamine Plus reagent measured by flow cytometry. Results are expressed as the mean percentage of RFP positive cells ± standard deviation. Results are expressed as mean measurements ± SD (n = 4). *Asterisks* represent LSD post hoc statistical significance compared to Lipofectamine Plus (P < 0.05)

**Fig. 3:**
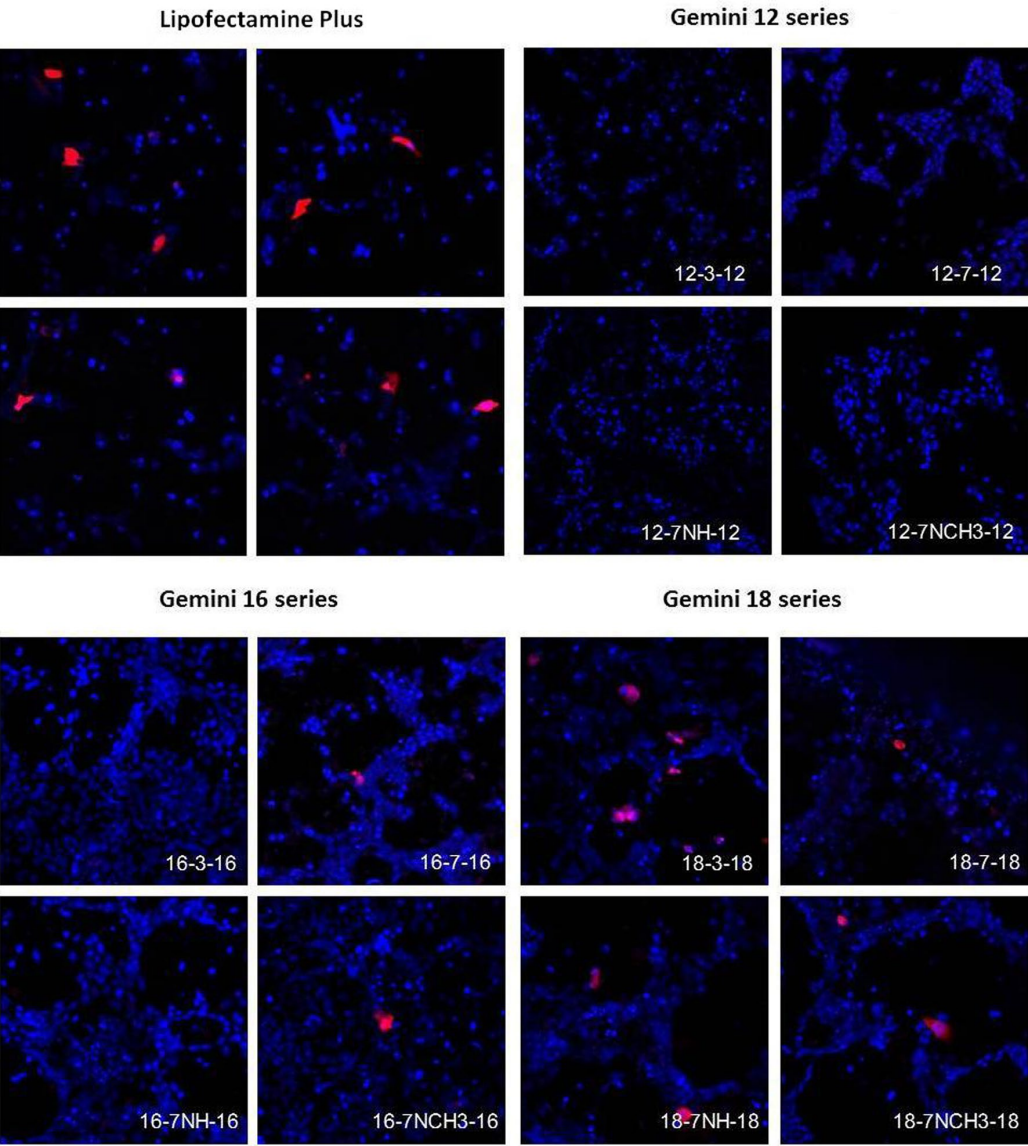
Confocal microscopic images of PAM212 cells treated with pDNA complexed to Lipofectamine Plus or GL-NPs, prepared using gemini surfactant series 12, 16, and 18. The expression of the tdTomato RFP is shown in *red* and nuclei were stained with DRAQ-5 and are shown in *blue*. Magnification ×20

**Fig. 4:**
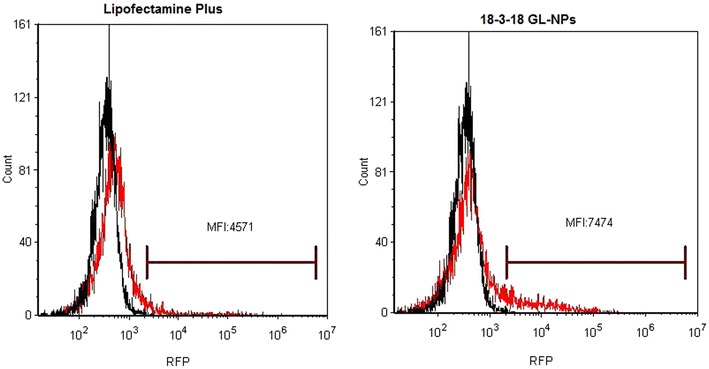
Median fluorescence intensity (MFI) of RFP positive PAM212 cells transfected by Lipofectamine Plus or 18-3-18 GL-NPs. MFI for RFP in cells transfected by 18-3-18 GL-NPs was 1.6-fold higher than MFI for RFP in Lipofectamine Plus treated cells. The *black* and *red peaks* represent control negative and test respectively

The expression of RFP in PAM212 keratinocytes transfected with the three series of GL-NPs carrying the tdTomato plasmid is shown in Fig. 2. GL-NP-mediated RFP expression in PAM212 cells was generally between 0 and 13 % and Lipofectamine Plus produced about 6 % RFP-positive cells. The 18-3-18 GL-NPs induced the highest RFP expression while RFP expression in GL-NPs from 12 and 16 series were significantly lower than Lipofectamine Plus (Fig. 2). The expression of RFP in PAM212 cells transfected with GL-NPs and Lipofectamine Plus were also confirmed by confocal microscopy (Fig. 3). The mean fluorescence intensity (MFI) of RFP expression after 18-3-18 GL-NPs transfection was 1.6 fold higher compared to Lipofectamine Plus (Fig. 4) indicating that not only transfection efficiency of GL-NPs was higher based on the number of cells transfected but also on the basis of intensity of gene expression (quantity of protein expressed).

### Flow cytometry analysis of cell membrane integrity and mitochondrial activity

**Fig. 5:**
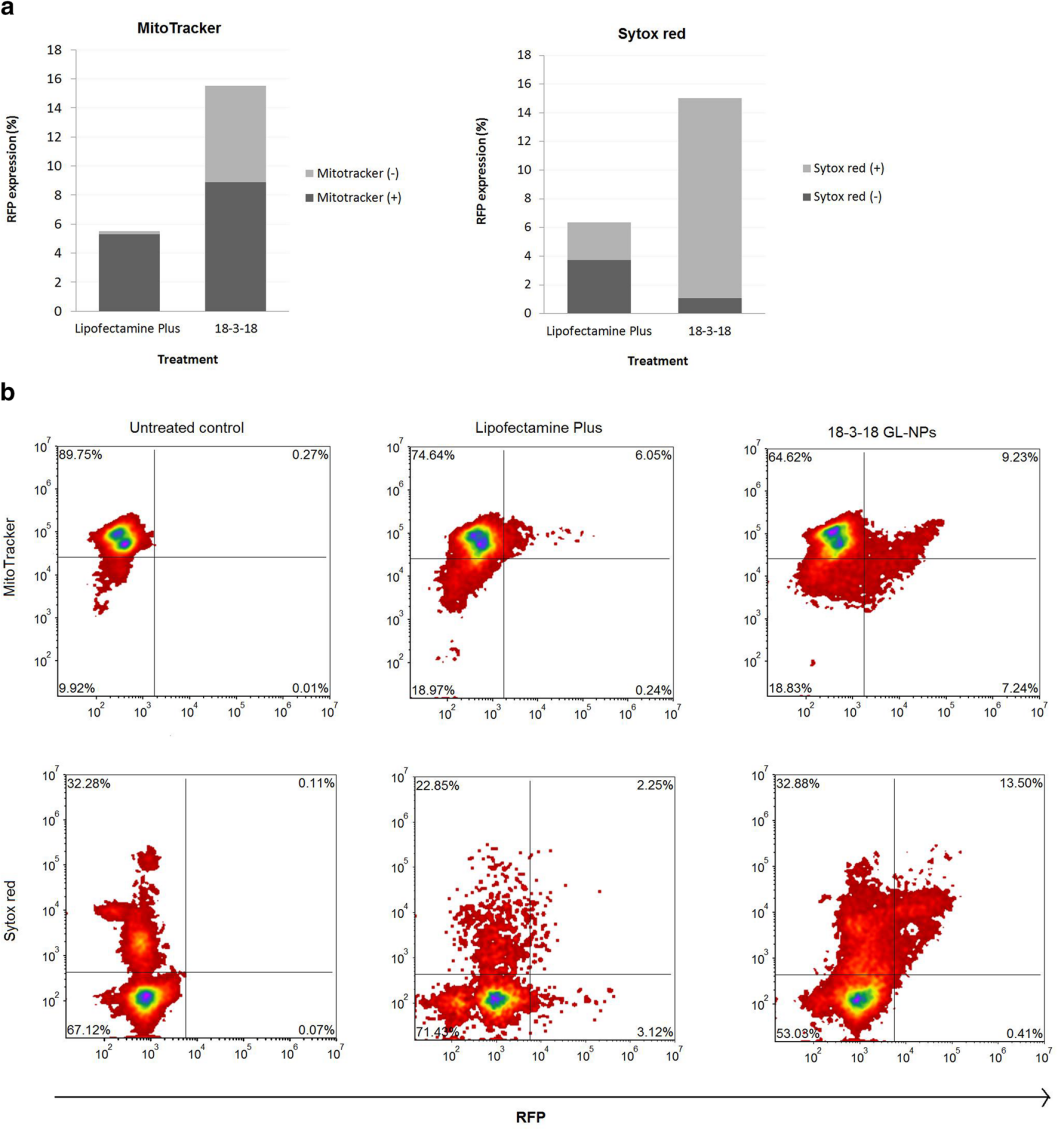
Flow cytometric analysis of RFP expressing PAM212 cells that were stained with either MitoTracker or Sytox red nucleic acid stain. Cells were transfected with either 18-3-18 GL-NPs or Lipofectamine Plus reagent. **a** A comparison between the proportion of live and dead PAM212 cells represented by either MitoTracker or Sytox *red* fluorescent signal. **b** The *density plots* show representative data from one of three separate experiments

In order to better understand the populations of cells expressing of RFP in PAM212 cells transfected with GL-NPs, we evaluated RFP expression in metabolically active (MitoTracker^+^) cells, or membrane-porated cells (Sytox red^+^) PAM212 cells by flow cytometry. Viability staining was performed at the same time on the same cell suspension sample that was divided into two microtubes and stained with MitoTracker Deep Red or Sytox red. In the case of GL-NP transfected cells, the presence of cell population that is both MitoTracker and Sytox red positive was an indication that cells could be alive while maintaining a compromised membrane. As shown in Fig. 5a, RFP expression was significantly higher in cells transfected with 18-3-18 GL-NPs compared to Lipofectamine Plus (15.5 vs 5.5 %); however, almost half of RFP positive cells (6.62 %) were considered as MitoTracker negative cells since they showed very low mitochondrial activity. Density plots in Fig. 5b also show that the majority of RFP positive cells were also positive for Sytox red (14.38 %), indicating that 18-3-18 GL-NPs disturbed cell membrane integrity, while cells were still alive and had mitochondrial activity.

**Fig. 6:**
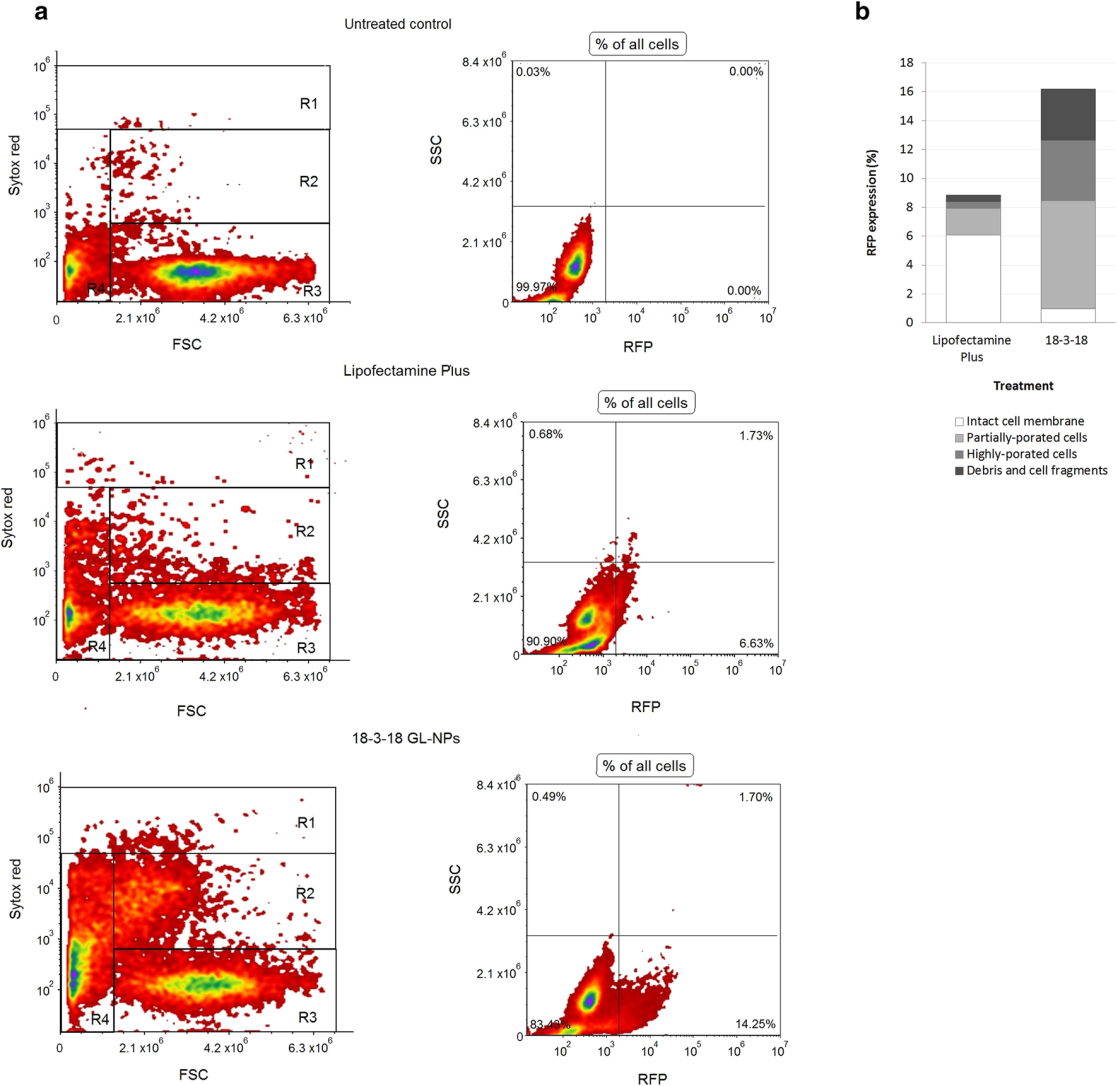
RFP expression in PAM212 cells with different status of cell membrane integrity. **a** The status of cell membrane integrity was determined based on the intensity of Sytox *red* DNA stain using flow cytometry; *R1* highly-porated cells, *R2* partially-porated cells, *R3* intact cells, *R4* debris and cell fragments. **b** *Stacked bar graph* presents the membrane status of cells expressing RFP after transfection by Lipofectamine Plus or 18-3-18 GL-NPs

Further analyses on the percentage of PAM212 cells with various stages of cell membrane integrity were performed using Sytox red staining. Sytox red is a high-affinity nucleic acid stain that penetrates into cells with disturbed plasma membranes, but will not cross uncompromised cell membranes. Cells with partially-porated membrane show increased Sytox red intensity, which is higher than those intact cells but lower as compared to cells with highly-porated membrane. These membrane permeability features help to separate different populations of PAM212 cells based on their Sytox red fluorescent intensity. As shown in Fig. 6a, cells were divided into four populations based on the size and the integrity of their membranes: intact membrane, partially-porated membrane, highly-porated membrane, and cell fragments. Results revealed that RFP expression in cells transfected with 18-3-18 GL-NPs was significantly higher in cells with partially- and highly-porated membranes, than Lipofectamine Plus-treated cells, which RFP was expressed mainly in intact cells (Fig. 6b). These results indicated that 18-3-18 GL-NPs were efficient transfection agents that affected cell membrane integrity. The cell population with partially-porated membrane was alive and MitoTracker positive, whereas cells with highly-porated membranes were dead and MitoTracker negative.

### Plasma membrane integrity and mitochondrial activity in PAM212 cells: a 3-color flow cytometry assay

**Fig. 7:**
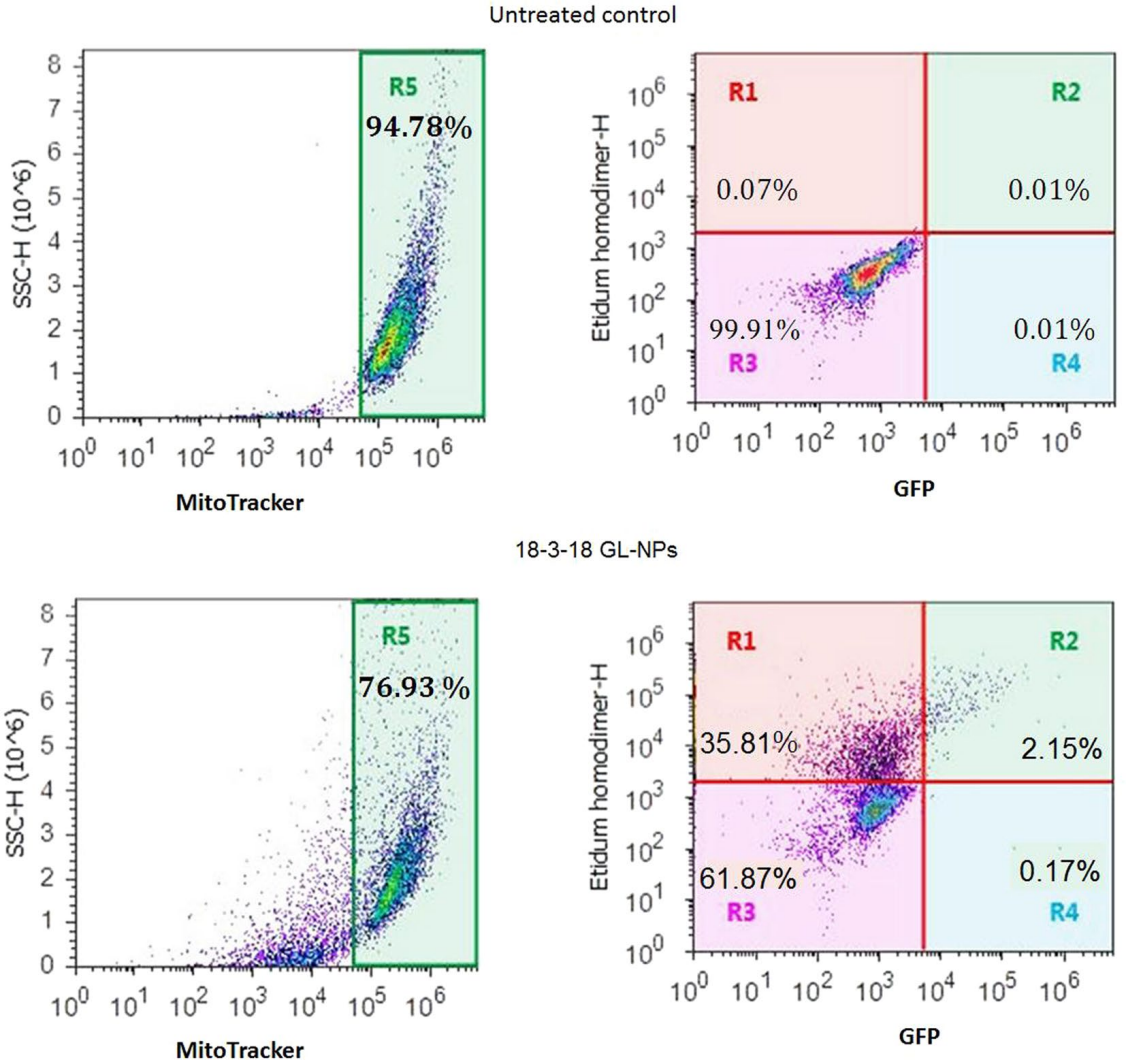
Flow cytometry *dot plot* of GFP expression in PAM212 cells, presenting partially-porated cells with active mitochondria but permeable cell membrane. All cells presented in the *right dot plots* (ethidium homodimer vs GFP) were in R5 gate and MitoTracker positive. Ethidium homodimer/MitoTracker double positive cells (partially-porated cells): R1 + R2, MitoTracker positive cells: R5, GFP positive cells: R4 + R2

Dual-parameter flow cytometry experiments with either Sytox red or MitoTracker stained RFP positive cells revealed the presence of more Sytox red positive cells than MitoTracker positive cells amongst the RFP expressing cell population, suggesting the existence of a subpopulation that is both viable and possesses a permeable membrane as it allowed the Sytox red to stain the nuclei. In order to further verify if the transfected cells with increased cell membrane porosity were still alive and positive for mitochondrial activity, a 3-color flow cytometry test was designed using GFP, MitoTracker and ethidium homodimer nucleic acid stain. Results in Fig. 7 revealed that 37.95 % (R1 + R2) of MitoTracker positive cells were also positive for ethidium homodimer in 18-3-18 GL-NPs transfected cells (Fig. 7).

### Analysis of GL-NPs uptake by light scatter

The intensities of FSC and SSC are proportional to the size of cells and the intracellular density, respectively. To clarify that the analysis of light scatters (FSC and SSC) using flow cytometry would be applicable for the evaluation of NPs uptake by PAM212 cells, the percentage of cells with higher SSC intensity were determined in Lipofectamine Plus or 18-3-18 GL-NP treated cells in comparison with untreated control.

**Fig. 8:**
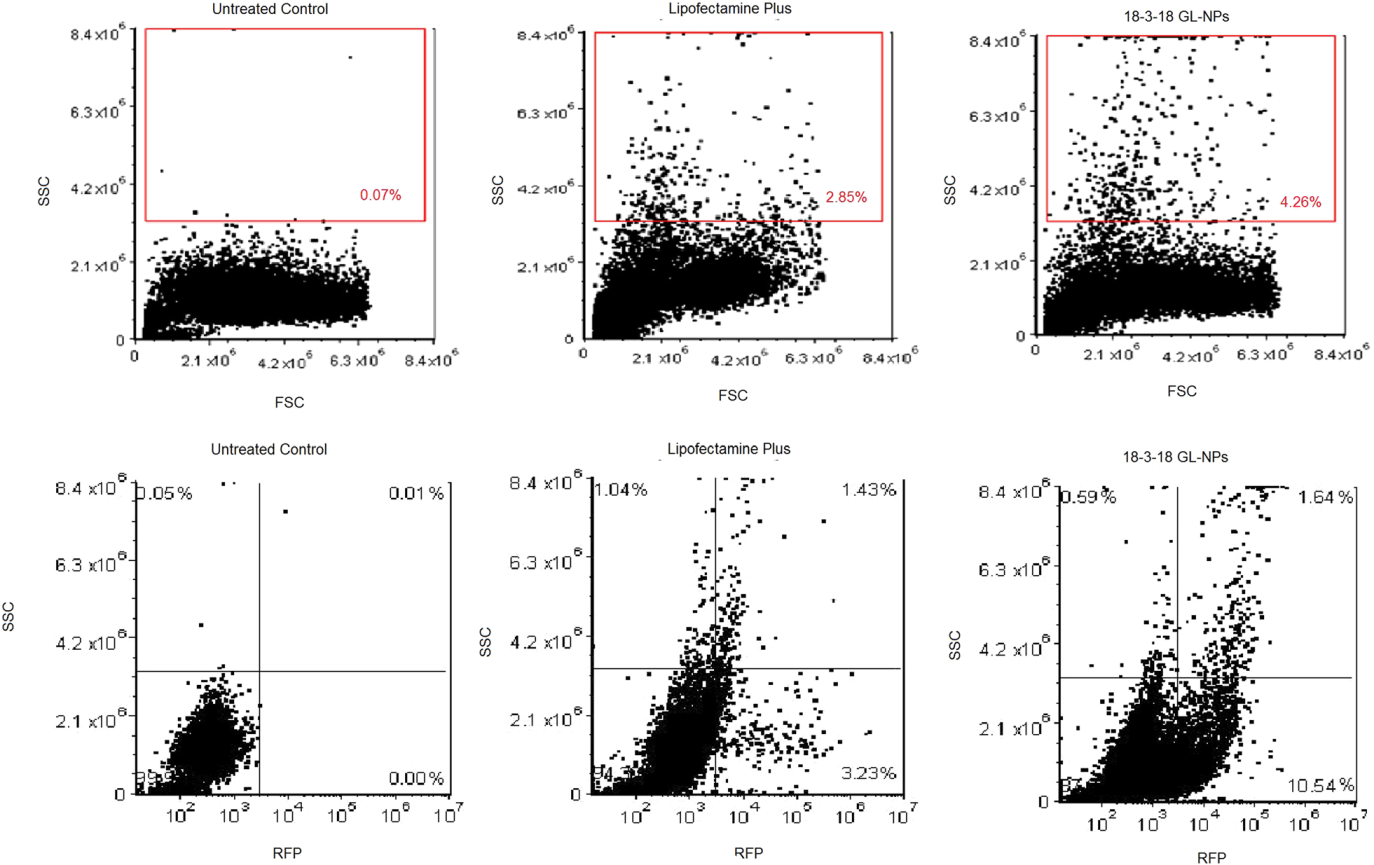
Evaluating SSC intensity as a parameter of NPs internalization into the cells using flow cytometry. **a** The SSC intensity of 18-3-18 GL-NPs treated cells was higher than Lipofectamine Plus treated and untreated cells (R1). **b** RFP expression in SSC high (R3) and low cell population (R4). Each *dot plot* represents 10,000 events

As shown in Fig. 8a, 4.26 % of cells treated with 18-3-18 GL-NPs had a significantly higher intensity of SSC than both the untreated control and the Lipofectamine Plus treated cells. The increase in SSC might be due to NP uptake and consequently the increase of cell granularity. Surprisingly, analyzing SSC intensity in those cells expressing RFP revealed that the majority of RFP positive cells transfected with 18-3-18 GL-NPs or Lipofectamine Plus (10.54 and 3.23 %, respectively), did not show high SSC intensity (Fig. 8b). This observation revealed that despite cellular uptake of NPs, as evidenced by RFP expression in the cells, intracellular density or granularity did not change significantly.

## Discussion

Transfection using GL-NPs constructed from tdTomato plasmid DNA, gemini surfactant, and a neutral lipid (DOPE) showed high transfection efficiency for the 18 series as well as 16-7-16 and 16-7NCH3-16 GL-NPs; whereas very low transfection was observed for the 12 series. The highest percentage of RFP positive cells was achieved using 18-3-18 GL-NPs, which was significantly higher (about twofold) than the commercial transfection reagent, Lipofectamine Plus (11.24 ± 4.7 vs 6.14 ± 3.01). Moreover, the MFI of RFP positive cells was significantly higher in 18-3-18 GL-NPs treatment than Lipofectamine Plus, indicating the brighter RFP expression level of each positive cells transfected by 18-3-18 GL-NPs. This finding is consistent with previous studies that have demonstrated alkyl tails longer than C12 are expected to encapsulate DNA more efficiently and result in both improved gene delivery and lower cytotoxicity [6]. In accordance with our previous observations [12], the incorporation of pH-sensitive aza and imino substituents within the spacer of gemini surfactants enhanced the transfection efficacy of m-7-m gemini surfactants, which can be related to pH-induced changes in NP structure that facilitates the fusion of NPs with endosomal membranes and release of DNA [12, 18].

Particle size was also evaluated for all GL-NPs and a very large particle size was observed in 18-3-18 GL-NPs compared to other gemini nanoparticles. Conflicting reports exist for the optimal size of lipoplexes for lipofection. Although some studies have reported high transfection efficiency for small particle size [19], our study showed superior transfection efficacy for 18-3-18 compared to other GL-NPs. Considering GL-NPs as cationic lipoplexes that bind to and permeating through the negatively charged cell membranes, the higher transfection efficacy of 18-3-18 large particles could be related to maximum contact with cells, which helps to increase phagocytic activity accompanied by better endosomal escape and cellular trafficking [7, 20].

Detailed analysis of 18-3-18 GL-NP transfected keratinocytes revealed that the permeability of cell membrane increased in cells that were otherwise viable after the transfection. We have also observed this in other studies where cells stained positive for both trypan blue and calcein AM, indicating that cells can show membrane disruption and be metabolically active simultaneously (results not shown). At this stage, the cells showed a slight loss of their membrane integrity, but still were alive and metabolically active, as conformed by MitoTracker staining in a multiparameter flow cytometry assay using GFP-transfected cells. We showed that that 37.95 % of the cells treated with 18-3-18 GL-NP were positive for both ethidium homodimer and MitoTracker, suggesting that NP treatment disturbed membrane integrity, while cells still had normal mitochondrial activity. This observation might be due to the physical interactions of the surface active positively-charged GL-NPs with the negatively-charged cell membrane, which lead to deformation and/or some degree of permeabilization, a type of ‘nanoporation’ of lipid bilayer membranes [4, 21].

Other studies have also described similar membrane-permeabilizing interactions of cationic NPs with synthetic and natural lipid bilayers. Leroueil et al. showed that positively charged dendrimers destabilized the cell membranes and were able to form holes, which led to dendrimer internalization into cells and diffusion of cytosolic proteins out of cells, and both intracellular and extracellular diffusion of dye molecules [15]. In another study by Chen et al. it has been found that non-cytotoxic concentrations of cationic NPs induce the formation of nanopores in lipid bilayers as well as natural cell membranes with total area from 1 to 350 nm^2^. That level of porosity caused leaking cytosolic proteins out of the cells, and fluorescent dyes such as fluorescein diacetate were able to pass through the cellular membrane and enter the cell [22]. The formation of holes on bilayer membranes has been also observed by cationic AuNPs in a vesicle-disruption assay, showing that cationic AuNPs were also able to disturb the lipid bilayer and release fluorescent dyes from vesicles [23]. In a coarse-grained molecular dynamics simulation that was done by Lin et al., it was found that cationic AuNPs generated nanopores in bilayers, which were expanded spontaneously within hundreds of nanoseconds. The initial hole appeared to be hydrophobic but it became hydrophilic as it grew larger (up to 10 nm of diameter), and allowed protein passage through the membrane [13]. In our flow cytometry experiments, based on the intensity of Sytox red DNA dye in the cells, two populations were observed: (1) cells with low Sytox red intensity, categorized as partially-porated cells and (2) cells with high Sytox red intensity as highly-porated cells. These two cell populations may represent different pore sizes in the cell membrane, caused by 18-3-18 GL-NPs.

Surprisingly, previous studies showed that the membrane permeability changes caused by nanostructures is not a static phenomenon, but is rather a dynamic process and membrane permeability could be reversible [24]. There is evidence that amphiphilic copolymers with intermediate hydrophobicity can perturb the lipid bilayer, translocate through membranes while significantly increase the permeability of membrane to water and small molecules [25]. However, there was no evidence for the formation of static structures like pores or holes in the cell membrane. Based on these reports together with our observation, it is reasonable to assume that the interaction of amphiphilic NPs such as GL-NPs with cell membrane facilitates its fusion with cell membrane, leading to membrane instability, which is not permanent and can be restored without affecting the longer-term viability of the cells.

Recently, Stefanutti et al. investigated the interaction of liposomes prepared from the 16-4-16 gemini surfactant bearing two methoxy groups in the spacer [(2*S*,3*S*)-2,3-dimethoxy-1,4-bis(*N*-hexadecyl-*N*,*N*-dimethylammonium)butane bromide] and dimyristoylphosphatidylcholine (DMPC) with fibroblasts using a combination of electro-rotation, atomic force microscopy and zeta potential measurements [26]. These tests showed that the gemini liposomes interacted with the cell membrane by changing its dielectric parameters without altering the membrane zeta potential, which was attributed to liposomes transversing the membrane without stable binding and accumulating in the cytoplasm as intact particles. According to the results of the MTT assay measuring metabolically active cells based on the reduction of 3-(4,5-dimethylthiazolyl)-2,5-diphenyltetrazolium bromide (MTT) by mitochondrial succinate dehydrogenase enzyme to formazan, the gemini liposome-exposed cells were considered 100 % viable, comparable to untreated cells. Interestingly, the observed decrease in capacitance C and conductance G and increase in cell radius by about 10 % was suggested to be the result of smoothing of the cell surface and flattening of membrane roughness [26].

The intensity of SSC parameter, as an indicator of cell granularity, has been recently used to detect nanoparticle internalization into the cells by flow cytometry [27–29] for metallic nanoparticles including TiO_2_ [29], ZnO, CuO, and Fe_3_O_4_ [28]. To gain further insight into the correlation between SSC intensity and cellular uptake of lipid-based nanoparticles, RFP expression in the cells transfected with 18-3-18 GL-NPs and Lipofectamine Plus was analyzed in SSC high and SSC low cell populations. Our results showed that in spite of NP uptake, the majority of cells expressing the RFP gene did not show high SSC intensity. This result suggests that an increase in SSC intensity may not be a certain proof for nanoparticle uptake since the visibility of nanoparticles in SSC depends on their size and density (refractive index). In this case, lipid-based NPs may have no effect on SSC intensity of the cells, whilst metallic nanoparticles due to their high refractive index might be easier to detect in the high SSC region of a flow cytometry dot plot.

## Conclusion

Taken together, 18-3-18 GL-NPs showed higher transfection efficiency and comparable viability profile to Lipofectamine Plus transfection reagent. We propose that the formation of cell membrane nanopores, without cytotoxic disrupting of the cell membrane integrity, could be a new mechanism of action for gene delivery by GL-NPs. These results have important implications for our understanding of the efficiency of GL-NPs as non-viral gene delivery agents in vivo. Further investigation of the molecular control of GL-NPs interaction with the cell membrane and the evaluation of the utilization of nanoscale-pore formation with high precision is warranted.

## Methods

### Gemini surfactants

**Fig. 9:**
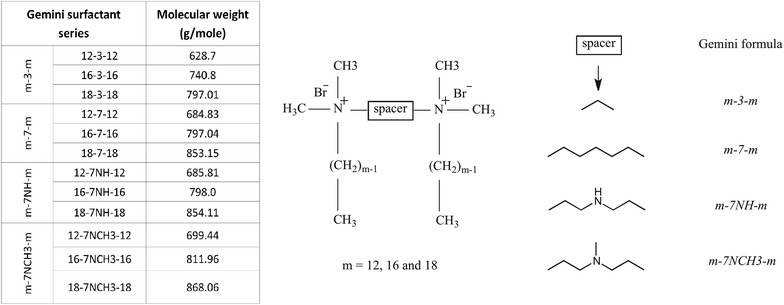
Chemical structure, formula and molecular weights of gemini surfactants in this study. *m* number of carbons in the hydrocarbon or alkyl tail, *s* number of methylene units in the unsubstituted spacer

Gemini surfactants (m-s-m) based on three different alkyl tail length series (m = 12, 16 and 18) were selected to make NPs (Fig. 9) and to investigate the effect of tail and spacer length, and functionalization on transfection efficacy and cell membrane integrity. Gemini surfactants were classified in four different groups based on the chemical structure of the spacer; gemini compounds with three or seven methylene unit spacers (m-3-m and m-7-m, respectively), pH-sensitive imino- or aza-substituted seven methylene-unit spacer groups (m-7NH-m and m-7NCH3-m, respectively). The synthesis of gemini surfactants were carried out as previously described [12, 30]. The commercially available Lipofectamine Plus (Life Technologies Inc. Rockville, MD, USA) was used as a positive control in each transfection experiment and prepared according to the manufacturer’s instructions.

### The preparation and characterization of GL-NPs

Gemini surfactants stock solutions were prepared at a final concentration of 300 µM in sterile, highly pure nuclease-free water (HyClone Laboratories, Logan, UT, USA). Aliquots were mixed with pCMV-tdTomato plasmid (pDNA, 0.5 μg) (Clontech Laboratories Inc., Mountain View, CA, USA) solution to obtain a pDNA: gemini surfactant charge ratio of 1:10 and incubated at room temperature for 15 min. Helper lipid vesicles (1 mM DOPE; Avanti Polar Lipids, Alabaster, AL, USA) were prepared in sucrose solution (9.25 % w/v) by bath sonication and high-pressure homogenization techniques using Microfluidizer LV1 (Microfluidics Inc., Newton, MA, USA) as described previously [10]. DOPE vesicles were added to the plasmid-gemini surfactant mixture (1:1 volume ratio) to form GL-NPs and incubated for 30 min at room temperature. The mean hydrodynamic diameters of the particles were measured with Zetasizer Nano ZS instrument (Malvern Instruments, Worcestershire, UK) equipped with a backscattered light detector operating at 173°. The homogeneity of NPs size distribution was recorded for each formulation as polydispersity index (PDI, range from 0 to 1).

### Transfection of PAM212 cells

Mouse PAM212 keratinocytes were maintained in minimum essential medium (MEM) supplemented with 10 % fetal bovine serum and 1 % penicillin/streptomycin. The cells were seeded 24 h prior to the transfection at 2 × 10^5^ cells/well in 24-well plates. The supplemented MEM was changed to basic MEM without serum and antibiotic 1 h prior to transfection. Keratinocytes in 200 µL basic medium were then incubated with 50 µL transfection medium containing either free plasmid (0.5 μg pDNA) or complexed with gemini-lipid or Lipofectamine plus reagents and incubated at 37 °C and 5 % CO_2_ for 5 h. Then, the cell medium containing nanoparticles was removed and replenished with 500 µL complete medium in each well. The expression of the tdTomato red fluorescent protein (RFP) was examined 19 h later by confocal microscopy (Zeiss LSM 710, Germany) and flow cytometry (Attune^®^ Flow Cytometer, Life Technologies, Carlsbad, CA, USA). To set up the flow cytometer parameters, high fluorescence RFP positive cells were prepared by electroporation of PAM212 cells with tdTomato plasmid (3 µg) using Keratinocyte Nucleofector Kit (VPD-1002, Lonza, Cologne, AG, USA) according to the manufacturer’s instructions.

### Confocal microscopy

The cells were seeded 24 h prior to the transfection at 8 × 10^5^ cells/well in six-well glass-bottom plates. The transfection was performed as mentioned previously and the expression of the tdTomato RFP was monitored 24 h after transfection. DRAQ-5 (Biostatus Ltd., Leicestershire, UK) was used as nuclear stain and images were captured using Zeiss LSM 710 inverted confocal microscope and Zen 2009 image analysis system (Carl Zeiss, Jena, Germany). To maintain consistency, all samples from a single experiment were stained in parallel, and images were acquired using the same settings.

### Evaluation of cell membrane integrity and mitochondrial activity by flow cytometry

The expression of the tdTomato RFP (excitation/emission ~554/581 nm) was determined 24 h after transfection. Each well was trypsinized and cells were transferred to microcentrifuge tubes. After centrifugation, the supernatant was removed and the pellet was resuspended in 1 mL PBS. The cell suspension was divided into two microtubes and stained with MitoTracker Deep Red (1 µM) mitochondrial stain for 20 min at 37 °C or Sytox Red (5 µM) dead cell stain for 5 min at room temperature in the dark. Transfection efficiency and cell viability were analyzed using Attune Acoustic Focusing Cytometer. RFP was excited with blue laser (488 nm) and detected in BL2 channel; while MitoTracker or Sytox red stains were excited with red laser (638 nm) and detected in RL1 channel. The intensity of Sytox red stain vs forward scatter (FSC) parameter was used to evaluate the status of cell membrane integrity. Viability was expressed as ‘cell viability index’ calculated according to the percentage of MitoTracker positive cells in untransfected wells as follows: (the percentage of live cells in each treated well/untransfected control) × 100, in which the viability index of control wells were considered as 100 %. Flow cytometry data was analyzed using Attune flow cytometer and FCS Express 4 Plus Software (De Novo Software, Los Angeles, CA, USA).

In order to analyze the effect of GL-NPs treatment on cell membrane integrity and metabolic activity of transfected cells, a three-color flow cytometry test was designed using green fluorescent protein (GFP, BL1 channel), MitoTracker Deep Red (RL1 channel), and Ethidium homodimer nucleic acid stain (excitation/emission ~528/617 nm; BL2 channel). Since RFP and Ethidium homodimer spectra overlapped and both were detected in BL2 channel, we used pCMV-GFP plasmid (gWiz-GFP plasmid; Aldevron, Fargo, ND; 5757 bp) instead of tdTomato plasmid (5392 bp) to make GL-NPs. GL-NPs preparation and cell transfection were done using the same conditions described earlier for tdTomato plasmid. GFP expression, metabolic activity and cell membrane permeability were analyzed using flow cytometry 24 h after transfection with 18-3-18 GL-NPs or Lipofectamine Plus reagent.

### Evaluation of NPs uptake by flow cytometry

In a flow cytometry light-scatter analysis, side scatter (SSC) reflects the inner density and the granularity within the cell. Based on this observation, previous studies have shown that the intensity of SSC would increase after NP uptake into the cells [27–29]. Therefore, in the present study, the cells in the high SSC region of the dot plots were analyzed to detect the uptake of GL-NPs and the expression of RFP gene in PAM212 cells. The 2D density plots of the SSC vs red fluorescence signals were created and the expressions of RFP protein in cell populations with high and low SSC intensity were analyzed using Attune cytometer and FCS Express 5 Plus (De Novo Software, CA, USA). A total of 10,000 events were counted per sample.

### Statistical analysis

Tests for significant differences between means of the transfection efficiency of the different formulations were carried out by One-Way ANOVA, followed by Fisher’s least significant difference (LSD) post hoc test using SPSS Statistics 21 (IBM SPSS, Chicago, IL, USA). Differences were considered statistically significant for P < 0.05.

## Abbreviations

GL-NPs: gemini surfactant-phospholipid nanoparticles
NPs: gemini nanoparticles
AuNPS: gold nanoparticles
DOPE: dioleoylphosphatidylethanolamine
RFP: red fluorescent protein
MFI: the mean fluorescence intensity
FSC: forward scatter
SSC: side scatter
MTT: 3-(4,5-dimethylthiazolyl)-2,5-diphenyltetrazolium bromide
